# Development of a Micro/Nano Probing System Using Double Elastic Mechanisms

**DOI:** 10.3390/s18124229

**Published:** 2018-12-02

**Authors:** Rui-Jun Li, Peng Xu, Peng-Yu Wang, Kuang-Chao Fan, Rong-Jun Cheng, Qiang-Xian Huang

**Affiliations:** 1 School of Instrument Science and Opto-electronics Engineering, Hefei University of Technology, Hefei 230009, China; pengxu@mail.hfut.edu.cn (P.X.); 2016110023@mail.hfut.edu.cn (P.-Y.W.); fan@dlut.edu.cn (K.-C.F.); chengrj@hfut.edu.cn (R.-J.C.); huangqx@hfut.edu.cn (Q.-X.H.); 2 School of Mechanical Engineering, Dalian University of Technology, Dalian 116024, China

**Keywords:** micro/nano CMM, probe, quadrant photodetector, elastic mechanisms

## Abstract

To meet the requirement of high precision measurement of coordinate measurement machine system, a compact microprobe has been designed for 3D measurement in this paper. Aiming to reduce the influences of signal coupling during the probing process, the probe has been designed by adopting two elastic mechanisms, in which the horizontal and vertical motions of the probe tip can be separated by differential signals of quadrant photodetectors in each elastic mechanism. A connecting rod has been designed to transfer the displacement of the probe tip in vertical direction from lower to upper elastic mechanisms. The sensitivity models in horizontal and vertical directions have been established, and the sensor sensitivity has been verified through experiments. Furthermore, the signal coupling of three axes has been analyzed, and mathematical models have been proposed for decoupling. The probing performance has been verified experimentally.

## 1. Introduction

In the past 20 years, microprobes have been in rapid development to meet the enormous demand of the precision engineering industry [1,2,3]. In particular, to characterize a 3D component with micrometer to millimeter scale features and requiring nanometer to submicron precision is required, such as micro holes, micro lens, micro fluid channels and other microcomponents with high aspect ratio or transparent material. As a versatile metrology tool, coordinate measuring machines (CMMs) with a microprobing system are extensively used as prospective measuring instruments. With regard to the coordinate metrology, the probe system is a critical element that limits the overall accuracy of a measurement.

Many microprobes have been proposed in the field of micro metrology. The first recognizable microprobe for CMMs was developed by PTB (Physikalisch-Technische Bundesanstalt, Germany) in 1998 [4]. This probe used an optoelectronic system as the sensing device. The design concept of using optical sensors was then adopted by many research institutions like Eindhoven University of Technology [5], Korea Advanced Institute of Science and Technology [6], the Nation Institute of Standards and Technology [7], Harbin Institute of Technology [8] and Interstate University of Applied Sciences and Technology [9]. The probe with optical sensors usually chose fiber stylus to detect the images observed by the ball tip. Most of the optical probes couldn’t fulfil the requirement of high accuracy, due to the fact that their measuring accuracy could be easily influenced by the surface or the material of the measured piece, and the pixels of the image processing instrument were also limited. High resolution could be also obtained by confocal sensor [10], quadrant photo detector [11], Fizeau interferometer [12], white light interference [13,14,15] and other complex photoelectric sensing technology in the probe design. However, the assembly of these sensors is costly and the volume of the assembled probes are usually bulky, which is not suitable for the measurement of micro devices.

Other highly sensitive sensing devices with high response speeds have been adopted in the probe design. PTB developed a microprobe on the basis of a microfabricated silicon membrane, whose position was measured using diffused piezo-resistive sensors [16]. The system allowed measurement forces in the range of few micro-Newtons. The horizontal and vertical stiffness was approximately 2 mN/µm and 48 mN/µm, respectively. The approximate design of the contact probe that used piezo-resistive sensors typically required an elastic element with methods of microsystem technologies, such as the probe designs from the Braunschweig University of Technology in Germany [17,18], Eindhoven University of Technology in Netherlands [19,20], and Tianjin University in China [21]. On the one hand, the stiffness of the suspensions in these probes is limited by the manufacturing tolerances, and it is difficult to achieve equal stiffness in all three axes in practice. On the other hand, the resolution can be improved, but the cost will be high. NPL (National Physical Laboratory, London, UK) developed a microprobe that had a light structure and low probing force [22,23]. The motion of the probe tip was sensed by capacitive sensors. The high resolution of capacitive sensors can be exploited in the probe design. However, nonlinearity emerges during the measuring process, and the measurement range is limited. METAS (Federal Institute of Metrology, Bern, Switzerland) adopted an alternative approach that exhibited perfectly isotropic probing forces through a monolithic spring box mechanism with flexure hinges [24,25]. The movement in each direction could be measured using an inductor sensor. The stiffness was 20 mN/mm, and the repeatability was approximately 5 nm. The detection principle by using a vibrating element as the sensing device could also be adopted in the probe design, such as the UMAP (Ultrasonic Micro and Accurate Probe) developed by Mitutoyo Corporation [26,27]. The repeatability of the probe was reported to be 0.1 µm, which became commercially available. The NPL also proposed a probe using a three-legged elastic mechanism with two piezo drives to generate vibrations [28,29]. Thus, the amplitude of vibrations could be sensed by a piezoelectric sensor. The contact force of the vibrating probe was much lower than other normal probes.

For ultraprecision measurement of micro devices with large aspect ratio, our group has previously developed several microprobes. The contact scanning probe based on four DVD (Digital Versatile Disc) pickup heads was conducted in the initial probe design [10]. The elastic mechanism of the four DVD pickup heads probe was composed of a wire-suspended floating plate and connected to a fiber stylus. Later, to reduce the size of four DVD pickup heads probe, a confocal sensor modified from the Blu-Ray pickup head was developed [30]. The developed probe based on the confocal sensor was verified to have a measurement resolution of 1 nm. However, the volume of the disc probe was still considerably bulky, and the measurement range was limited by the utilized focus sensor. To reduce the size of the probe and expand the measurement range, a 2D angle sensor modified from a DVD pickup head and a miniature Michelson interferometer was developed for the probing system to measure the angle and displacement of the probe [31]. In several experiments, we found difficulties in maintaining a uniform tension of the suspending wires during the assembly. This problem can be solved by replacing the spring wire with a patterned leaf spring made of beryllium bronze sheet, which can yield favorable reproducibility [32]. However, the horizontal scanning range of this newly probe is limited due to the separation of two interfered beam spots and the decrease of fringe intensity when the probe is touched in the horizontal direction. In order to expand the measurement range, a parallel optical path was adopted using two separate laser diodes [33]. This new parallel optical path probe could reach a measurement range of ±20 μm both in horizontal and vertical directions. Then, we used a quadrant photodetector (QPD) as the sensing device to simplify the light path [34]. However, the adopted signal sensor cannot distinguish the different signals between the horizontal and vertical directions. And the sensitivity of the single sensor probe in vertical direction is lower than horizontal direction. The single sensor probe can only be used as a touch trigger probe. Aiming to increase the functionality and the practicality of the probe, a new structure design for the 3D contact scanning probe is conducted to increase the accuracy of the measurement results.

In this study, the probe principle based on double elastic mechanisms is proposed. The probe can reduce the signal coupling of three axes by structure design. The sensitivity model in the horizontal and vertical directions has been established to estimate the sensitivity of the sensor. The coupling of three axes has been analyzed, and the mathematic models have been proposed for decoupling. Furthermore, the probing performance has been verified through experiments.

## 2. Probe Principle and Design

### 2.1. Principle of the Microprobe

**Figure 1:**
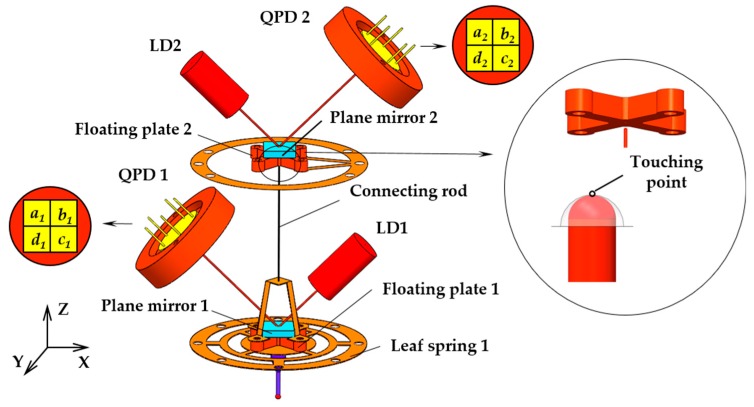
Structure of the probe.

**Figure 2:**
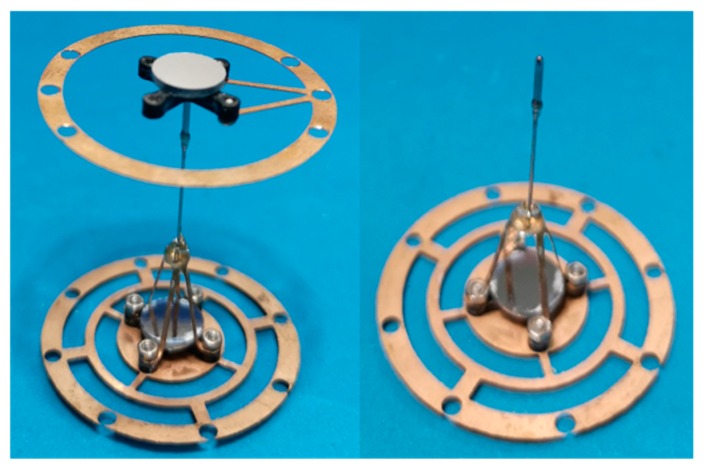
Photo of the elastic mechanisms and the rod.

**Figure 3:**
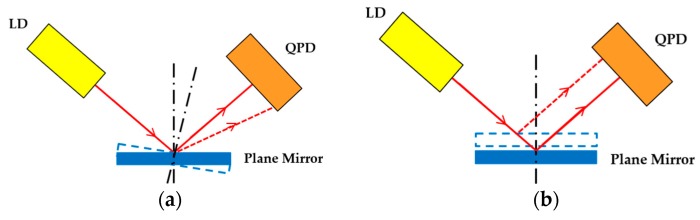
Sensing principle of the probe when the probe tip has a displacement (**a**) in horizontal (**b**) in vertical.

The microprobe system is composed of two same optoelectronic sensors, two elastic mechanisms and a stylus, as shown in Figure 1. Each sensor consists of a laser diode (LD), a plane mirror and a quadrant photo detector (QPD). Each elastic mechanism consists of a patterned leaf spring and a floating plate with a plane mirror. A slender rod was designed to connect the two elastic mechanisms. The upper end of the connecting rod is designed as a hemispherical structure, so as to support the floating plate 2 in a non-constraint and point-contact way. The lower end of the connecting rod is installed on the floating plate 1 by a hollow frame in order to prevent blocking the light from LD 1. Figure 2 shows the photo of the elastic mechanisms and the rod. A laser beam from LD 1 is reflected by the plane mirror 1 in the lower elastic mechanism. The reflected beam will project on the photo surface of QPD 1. When a contact force is applied to the probe tip in horizontal, only the leaf springs 1 in the lower elastic mechanism will undergo an elastic deformation and then only the plane mirror 1 will have an angle along lateral axes. This motion will be simultaneously detected by QPD 1. Figure 3a depicts the sensing principle of the probe in this case. The light intensity detected by QPD 1 is converted into two voltage signals corresponding to the probe tip’s displacements in *x* and *y* directions (probe coordinate system). The two voltage signals are defined in Equations (1) and (2), respectively.

(1) $$U_{x}=k_{x}\times [(P_{a1}+P_{b1})-(P_{c1}+P_{d1})],$$

(2) $$U_{y}=k_{y}\times [(P_{a1}+P_{d1})-(P_{b1}+P_{c1})],$$

where $P_{a1}$, $P_{b1}$, $P_{c1}$, $P_{d1}$ are the optical powers detected by the four photodiodes of QPD 1 respectively. $k_{x}$, $k_{y}$ are constants.

Similarly, when a contact force is applied in vertical, the action on the probe tip will be transferred to the upper elastic mechanism through the connecting rod. The floating plate 1 will only have a translation in vertical since its angular displacement is limited by the centrally symmetrical leaf spring 1; the *x* signal from QPD 1 will have a new output that is a cross-talk error and can be reduced by the decoupling method in Section 4. The floating plate 2 will have both a translation in vertical and an angular displacement, the signal from QPD 2 corresponding to the $u_{x}$ signal of QPD1 will also have a new output, which is used as the sensing signal in vertical, the sensing principle is shown in Figure 3. Therefore, the voltage signal corresponding to probe tip’s displacement in *z* direction can be defined as follows:

(3) $$U_{z}=k_{z}\times [(P_{a2}+P_{b2})-(P_{c2}+P_{d2})].$$

where $P_{a2}$, $P_{b2}$, $P_{c2}$, $P_{d2}$ are the optical powers detected by the four photodiodes of QPD 2 respectively. $k_{z}$ is a constant.

### 2.2. Uniform Stiffness

**Figure 4:**
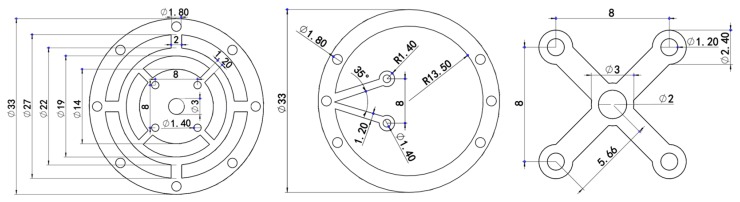
Dimensions of the leaf spring 1 (**left**), leaf spring 2 (**middle**) and the floating plate 1 and 2 (**right**), (unit: mm).

**Figure 5:**
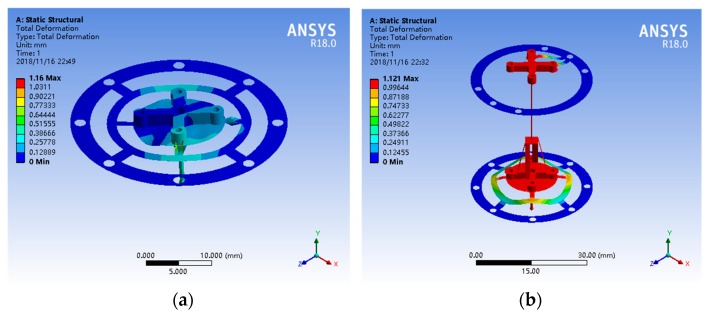
Simulation results when a 1 mN force is applied to the probe tip: (**a**) in horizontal; (**b**) in vertical.

**Figure 6:**
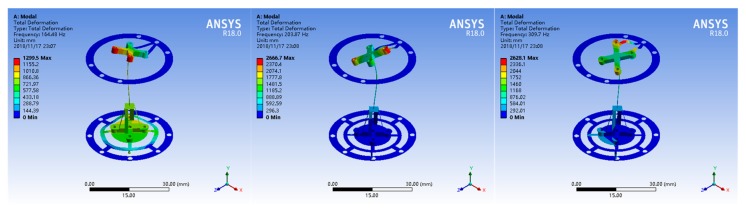
Mode analysis results of the probe: first order (**left**), second order (**middle**), third order (**right**).

The stiffness of the probe should be isotropic and controlled to less than 1 mN/μm [31]. To obtain an appropriate stiffness, two patterned leaf springs and floating plates as Figure 1 and Figure 2 shows were designed. Simulation analysis using ANSYS software was performed to design and optimized the elastic mechanisms since it is difficult to calculate the mechanical model of the complex leaf spring. Several parameters of the elastic mechanism affect the stiffness of the probe directly, such as material, diameters, widths, thickness and the length of the stylus. Beryllium bronze was used to fabricate the leaf springs because of its excellent elasticity. According to the specification of the selling stylus, the length of the stylus was determined as 10 mm. The remained parameters were tried, analyzed and optimized repeatedly using ANSYS software until achieving a uniform stiffness of less than 1 mN/μm in 3D. Most of the appropriate parameters of the elastic mechanisms are shown in Figure 4 and the others are listed in Table 1. The simulation results using the optimized parameters are shown in Figure 5. From Figure 5, we can see that the probe tip will have a displacement of 1.16 μm and 1.12 μm in horizontal and vertical respectively when a 1 mN force is applied. The simulation results (Figure 5 shows) indicate that the probe tip has a uniform stiffness of 0.86 mN/μm in lateral directions and a quite consistent stiffness of 0.89 mN/μm in vertical direction. Mode analysis was also conducted by ANSYS software to investigate the dynamic characteristics of the probe, the results are shown in Figure 6. The first order natural frequency of the probe should be high than 60 Hz [31]. From Figure 6 we can find that the natural frequencies of the probe are 164.5 Hz (first order), 203.9 Hz (second order), 309.7 Hz (third order) respectively, which is high enough for the probe.

### 2.3. Sensitivity Analysis

**Figure 7:**
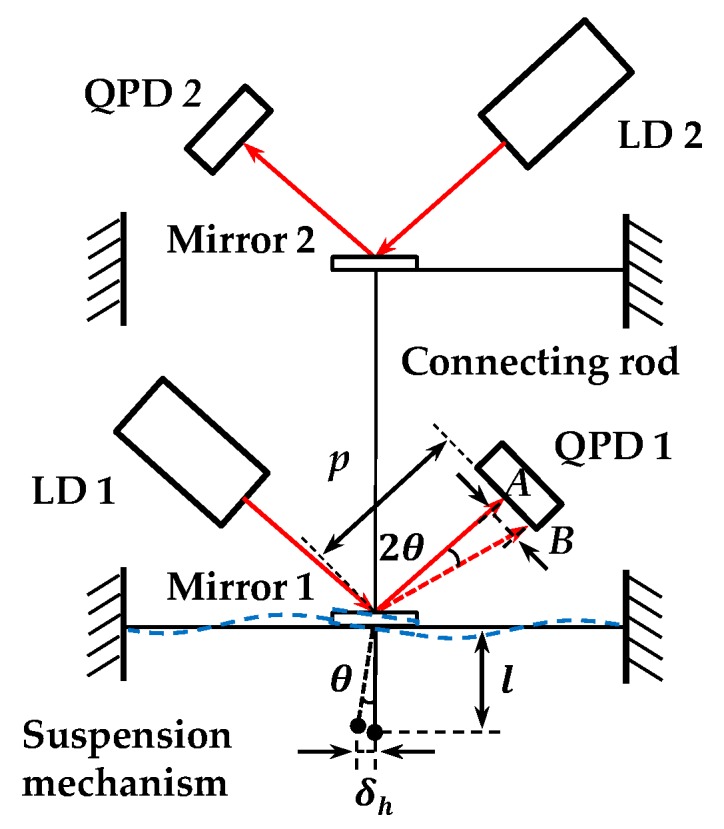
Sensing principle in horizontal direction.

In the sensing unit, the reflected beam will project on the QPD at the center of the active area. The displacement of the light spot induced by the mirror deflection can be detected when a contact force is applied. Figure 7 exhibits the detecting principle in horizontal. The upper elastic mechanism is not affected by the probe tip’s motion in this case, because the slender rod supports the floating plate 2 in a non-constraint and point-contact way and θ is smaller than 7.2″ ($\delta_{h}$ < 20 μm, *l* = 10 mm). We can obtain that the caused vertical displacement of floating plate 2 is less than 1 nm when the probe tip is pushed 20 μm in horizontal. The light beam with an incident angle is reflected by the plane mirror 1 and focused onto the surface of the QPD 1. The focused spot is shifted from point A to point B on the surface of the QPD 1. From the geometrical relationship of the light path, we have:

(4) $$\bar{AB}=p\times \tan2\theta ,$$

(5) $$\sin\theta =\delta_{h}/l,$$

where *p* is the distance between the QPD surface and the reflecting point on the plane mirror; *l* is the length of the stylus. The output voltage $U_{h}$ is proportional to the displacement of the focused spot and $U_{h}=k_{h}\times \bar{AB}$ ($k_{h}$ is the constant and can be calibrated). A relationship between displacement $\delta_{h}$ and sensitivity $S_{h}$ can be described by the following equation:

(6) $$S_{h}=\frac{U_{h}}{\delta_{h}},$$

Because θ is a considerably small angle, the approximations sinθ≈θ, tan2θ≈2θ can be made, then $S_{h}$ can be described as follows:

(7) $$S_{h}=\frac{U_{h}}{\delta_{h}}=k_{h}\times \frac{p\times \tan2\theta }{l\times \sin\theta }\approx 2k_{h}\times \frac{p}{l},$$

**Figure 8:**
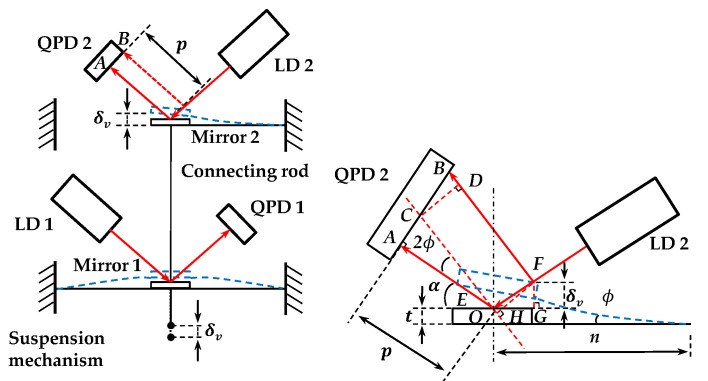
Sensing principle in vertical direction.

Similarly, Figure 8 illustrates the deflection and sensing principle of the probe when a contact force is applied along vertical direction. Mirror 2 has a translation in vertical and an angular displacement, which cause an optical spot shift of $\bar{CB}$ and $\bar{AC}$. With respect to the geometry depicted in Figure 8, we can obtain the total shift of the optical spot $\bar{AB}=\bar{AC}+\bar{CB}$. In ΔOAC and ΔBCD, we have:

(8) AC=p×tan2θ,

(9) $$\bar{CB}=\bar{CD}/\cos2\theta =\bar{FH}/\cos2\theta ,$$

From the geometrical relationship of the optical path, we can derive $\bar{FG}\approx \delta_{v}\approx n\times \tan\phi$ (because ϕ is considerably small); thus, in ΔOFH and ΔOFG, it can be obtained that:

(10) $$\bar{FH}=\bar{OF}\times \sin[\pi -(\pi -2\alpha -2\phi )]=\bar{OF}\times \sin2(\alpha +\phi ),$$

(11) $$\bar{OF}=\frac{\bar{FG}}{\sin\alpha }=\frac{\delta_{v}}{\sin\alpha },$$

Here, α is the angle between incident light and plane mirror. Because all the reflect angles are small in practice and $U_{v}=k_{v}\times \bar{AB}$, the displacement $\delta_{v}$ and sensitivity $S_{v}$ can be written as

(12) $$S_{v}=\frac{U_{v}}{\delta_{v}}=\frac{k_{v}\times [p\times \tan2\phi +\frac{\delta_{v}\times \sin(2\alpha +2\phi )}{\sin\alpha \times \cos2\phi }]}{\delta_{v}},$$

Since ϕ is small, α+ϕ≈α, cos2ϕ≈1, $\tan\phi \approx \phi \approx \delta_{v}/n$ (*n* is the distance from the reflected point of the light to the fixed point of the leaf spring). Equation (12) can be expressed as:

(13) $$S_{v}=\frac{U_{v}}{\delta_{v}}\approx 2k_{v}\times (\frac{m}{n}+\cos\alpha ),$$

Let Equation (7) is considered equal to Equation (13) to determine the uniform sensitivity, thus yielding

(14) $$\cos\alpha =\frac{p}{l}-\frac{p}{n}.$$

On the basis of the constrained conditions of uniform sensitivity and stiffness in three dimensions, the remained parameters have also been optimized as α=45°, l=10 mm, t=1 mm, n=14 mm, and p=24 mm.

## 3. Experiments

**Figure 9:**
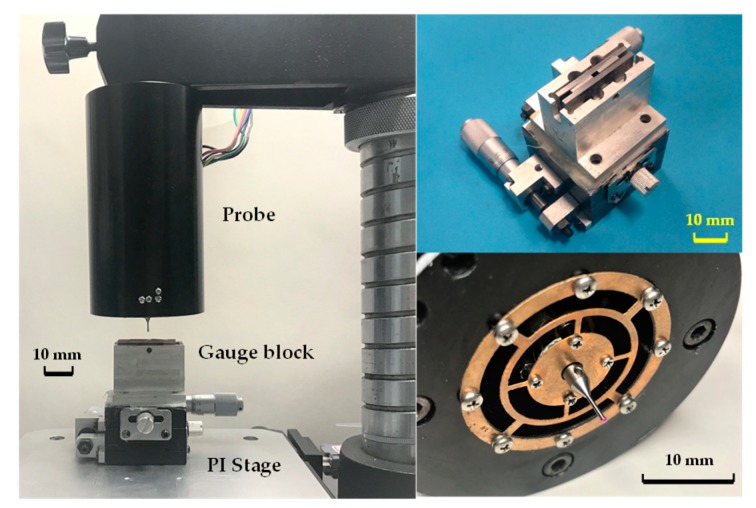
The probe and experimental setup.

Figure 9 shows the photos of the probe and an experimental setup to investigate the probe’s performance. The probe was assembled in a frame of a stand. A 2 mm × 2 mm square hole formed by four 0 grade gauge blocks was used to contact the probe tip in lateral directions. The thicknesses of the gauge blocks are 2 mm, 2 mm, 1.6 mm and 1.7 mm respectively. The gauge blocks with 2 mm thickness are clamped by the other two. Two parallel surfaces of square hole are the reference planes of the gauge block, but the other two are not. Therefore, the probe tip can be contacted in a lateral direction by the two reference planes of the square hole. The contacting direction can be changed by rotating the shaft of the probe, assisting with a round dial with an accuracy of 0.5 degree. A 2D linear stage was used to change the initial position of the square hole manually. A high-precision 3D nano-positioning stage (Physik Instrument (PI), model P561.3 CD with 2 nm repeatability and 100 μm travel, Karlsruhe, Germany) was used as a displacement reference.

### 3.1. Drift

**Figure 10:**
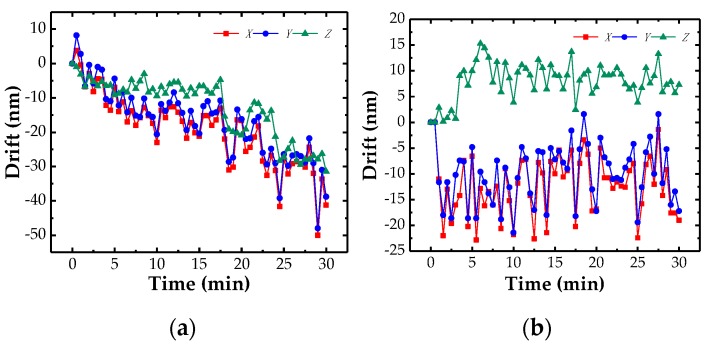
Drifts of the probe: (**a**) the temperature was not controlled; (**b**) the temperature was controlled.

**Figure 11:**
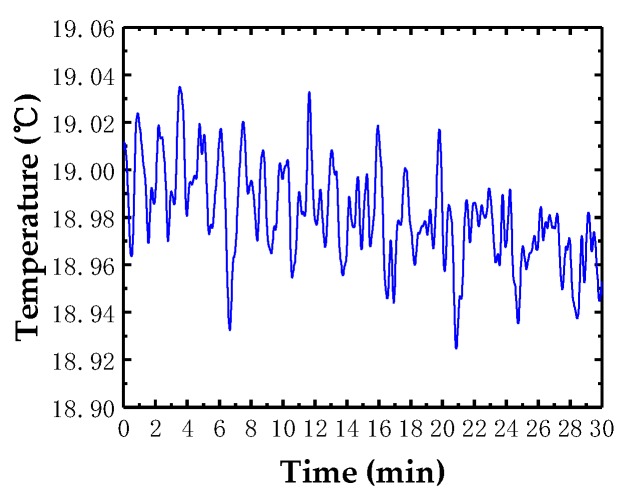
Temperature in the chamber.

Stability is a key parameter of the probe, which needs to be tested. Considering that most of the components can be measured within half an hour, the drift of the probe was tested about 30 min in this study. Figure 10a depicted the drift of the probe in 30 min when the temperature was not controlled. The drift reaches about 60 nm, which caused by the temperature fluctuation. A similar test was also conducted when the probe was covered by a constant temperature chamber developed by ourselves [35]. Figure 10b demonstrated the results when the temperature’s fluctuation was controlled within less than 0.13 °C (Figure 11 shows). The drift is approximately 23 nm in 30 min, which can be reduced furthermore if a more stable temperature was achieved.

### 3.2. Repeatability

**Figure 12:**
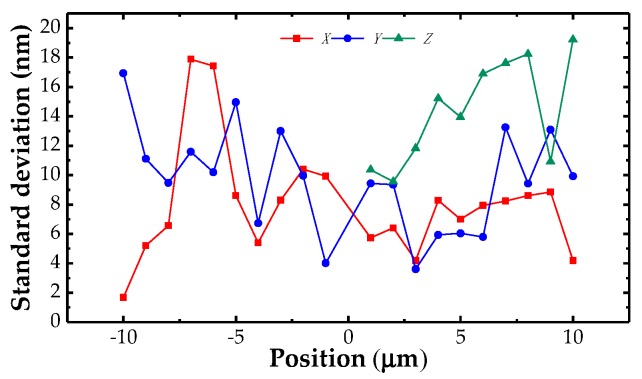
Results of the repeatability measurement.

The behavior of single-point probing was also tested through the experimental setup as Figure 9 shows. The probe tip was contacted by the reference planes of the high-accuracy gauge blocks driven by the PI stage along the *X+*, *X−*, *Y+*, *Y−*, and *Z* directions respectively. For each touching action, the probe tip was pushed 1 µm, the output signals were recorded and then the gauge blocks were moved back. The procedure was repeated 10 times in each point. Figure 12 exhibited the repeatability measurement results of the probe. The maximum standard deviation is 18.2 nm within the measurement range of ±10×±10×10 µm (*X* × *Y* × *Z*).

### 3.3. Resolution

**Figure 13:**
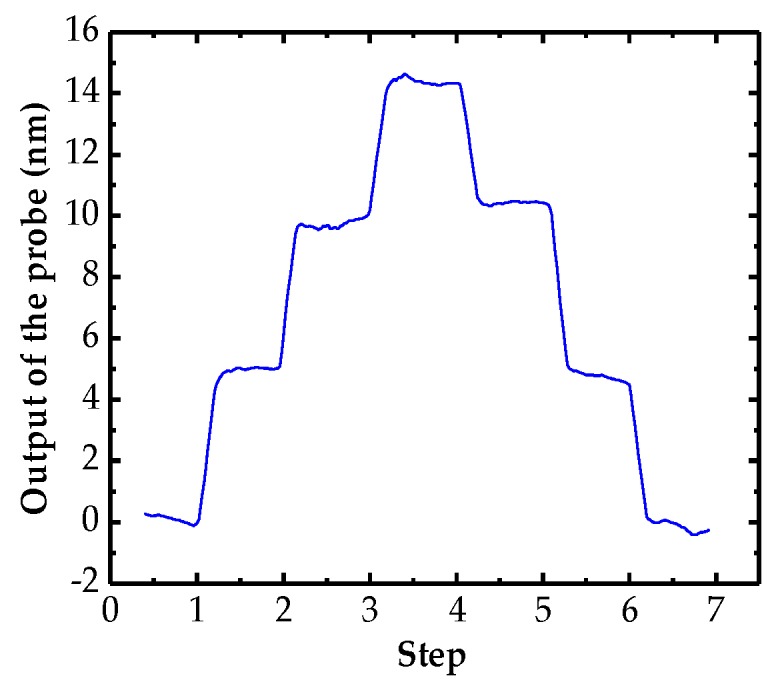
Results of the resolution test for the probe.

The resolution of the probe in its measurement range is an important parameter that determines the precision of the whole CMM system. An experiment was conducted to further verify the measurement resolution of the probe by using the experimental setup as Figure 9 shows. At the beginning of the experiment, the stage was controlled to drive the gauge block to push the probe tip and then reverted to the initial position. The probe was pushed forward three steps and then retracted. The moving step was set from 10 nm to 5 nm with an interval of 1 nm in sequence and the resolution could be determined by the minimum clear step. Figure 13 illustrates that the resolution of the probing system is better than 5 nm.

## 4. Decoupling of the Cross-Talk Errors

Cross-talk errors usually exist because of the misalignment between probe coordinate systems and CMM stage coordinate systems, which should be calibrated and decoupled. Multiple regression methods can be applied and a transfer matrix can be derived to transform the outputs of the probe ($U_{x}$, $U_{y}$, $U_{z}$) to the displacements of the probe tip (*X*, *Y*, *Z*) in a CMM stage coordinate system [36].

(15) $$\left(\begin{array}{ccc} X_{1} & Y_{1} & Z_{1}\\ X_{2} & Y_{2} & Z_{2}\\ \vdots & \vdots & \vdots \\ X_{n} & Y_{n} & Z_{n} \end{array}\right)=\left(\begin{array}{cccc} U_{x1} & U_{y1} & U_{z1} & 1\\ U_{x2} & U_{y2} & U_{z2} & 1\\ \vdots & \vdots & \vdots & \vdots \\ U_{xn} & U_{yn} & U_{zn} & 1 \end{array}\right)\left(\begin{array}{ccc} a_{1} & a_{2} & a_{3}\\ b_{1} & b_{2} & b_{3}\\ c_{1} & c_{2} & c_{3}\\ d_{1} & d_{2} & d_{3} \end{array}\right),$$

**Figure 14:**
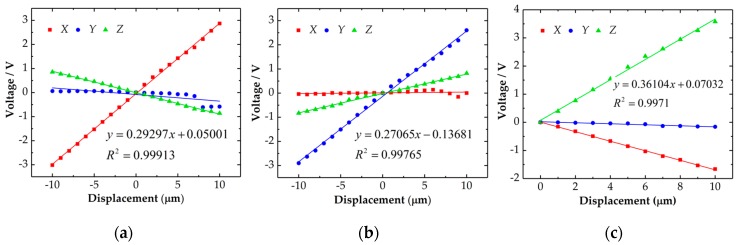
Test results of the probe in *X* (**a**), *Y* (**b**) and *Z* (**c**) direction.

In order to calculate the transfer matrix, a test was conducted in *X+*, *X−*, *Y+*, *Y−* and *Z* direction respectively with an interval of 1 μm. The experimental setup as Figure 9 shows and a similar measuring process of repeatability test was also used. In order to minimize the influence of the random errors, 10 tests were repeated in each position and the average values shown in Figure 14 were used. Figure 14 shows that the probe has a basically uniform sensitivity of 0.3 V/μm. The difference could be caused by the manufacturing and assembling errors.

Substituting the test data shown in Figure 14 into Equation (15), we can obtain the following transfer matrix by applying the least-squares methods.

(16) $${\hat{X}}_{0}=\left(\begin{array}{ccc} a_{10} & a_{20} & a_{30}\\ b_{10} & b_{20} & b_{30}\\ c_{10} & c_{20} & c_{30}\\ d_{10} & d_{20} & d_{30} \end{array}\right)=\left(\begin{array}{ccc} 2.44965 & 0.032749 & -1.10654\\ -2.41337 & 3.002049 & 4.181964\\ 1.199704 & -0.09759 & -2.06242\\ -0.0019 & 0.003539 & -0.1431 \end{array}\right),$$

The accuracy of the estimated values can also be obtained from the error matrix obtained by the least-squares method [37]:

$$\sigma =\left(\begin{array}{ccc} 5.1 & 4.0 & 6.2\\ 5.3 & 4.1 & 6.3\\ 4.1 & 3.2 & 4.9\\ 5.9 & 4.6 & 7.1 \end{array}\right)$$

**Figure 15:**
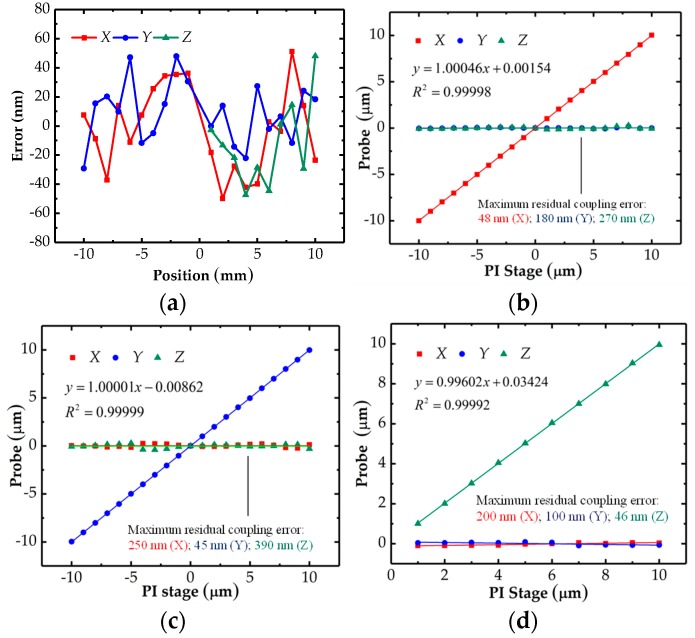
Results of the decoupling process: (**a**) residual errors; (**b**) in X direction; (**c**) in Y direction; (**d**) in Z direction.

The above calibration experiments were conducted again to verify the effectiveness of the obtained transfer matrix. The probe was tested one time in each position. The errors of the decoupling model in each axis shown in Figure 15a can be obtained by comparing the displacements of the PI stage with the values calculated by the transfer matrix. Figure 15a illustrates that the probe error caused by the decoupling model was less than ±50 nm in each direction. Figure 15b–d presents the decoupled results of the probe when the probe was contacted along *X*, *Y* and *Z* directions respectively. The cross-talk errors have been reduced from several micrometers to 390 nm. The nonlinearity of the line *X* in Figure 15b, line *Y* in Figure 15c and line *Z* in Figure 15d are 0.28%, 0.19% and 0.42% respectively.

## 5. Conclusions

This study presents an innovative probe with low interference between horizontal and vertical measurement by adopting two elastic mechanisms. A connecting rod with a hemispherical end was used to transfer vertical displacement of the probe tip to the upper elastic mechanism. The horizontal motions of the probe tip can only be detected by the lower elastic mechanism and its sensor, while the upper elastic mechanism and its sensor can only detect the vertical motions of the probe tip. The probe has been designed, analyzed and optimized. The simulation results verified that the probe has a uniform stiffness in 3D. The experimental results show that the probe has a repeatability of 20 nm, a resolution of 5 nm, an analog measurement range of larger than ±10 μm, and a stability of 23 nm within 30 min when the fluctuation of the environment temperature was controlled within 0.13 °C. The crosstalk errors of the probe have been compensated. The probe can be used in the coordinate measurement machine and any other precision measurement systems. A reference sphere will be tested by the developed probe to evaluate its 3D accuracy in future.

## Figures and Tables

**Table 1:** Parameters of the probe.

Parameters	Value
Material of the leaf spring	Beryllium–copper alloy
Young’s modulus of the leaf spring (Gpa)	130
Material of the floating plate	Aluminum alloy
Weight of the floating plate (g)	1.5
Thickness (mm)	2
Young’s modulus of the floating plate (Gpa)	71
Material of the stylus	Tungsten stylus with a ruby ball tip
Length of the stylus (mm)	10
Diameter of the probe ball (mm)	1
Young’s modulus of the stylus (Gpa)	193
Distance between the two leaf springs (mm)	34
